# Ten Simple Rules for Doing Your Best Research, According to Hamming

**DOI:** 10.1371/journal.pcbi.0030213

**Published:** 2007-10-26

**Authors:** Thomas C Erren, Paul Cullen, Michael Erren, Philip E Bourne

This editorial can be considered the preface to the “Ten Simple Rules” series [1–7]. The rules presented here are somewhat philosophical and behavioural rather than concrete suggestions for how to tackle a particular scientific professional activity such as writing a paper or a grant. The thoughts presented are not our own; rather, we condense and annotate some excellent and timeless suggestions made by the mathematician Richard Hamming two decades ago on how to do “first-class research” [8]. As far as we know, the transcript of the Bell Communications Research Colloquium Seminar provided by Dr. Kaiser [8] was never formally published, so that Dr. Hamming's thoughts are not as widely known as they deserve to be. By distilling these thoughts into something that can be thought of as “Ten Simple Rules,” we hope to bring these ideas to broader attention.

Hamming's 1986 talk was remarkable. In “You and Your Research,” he addressed the question: How can scientists do great research, i.e., Nobel-Prize-type work? His insights were based on more than forty years of research as a pioneer of computer science and telecommunications who had the privilege of interacting with such luminaries as the physicists Richard Feynman, Enrico Fermi, Edward Teller, Robert Oppenheimer, Hans Bethe, and Walter Brattain, with Claude Shannon, “the father of information theory,” and with the statistician John Tukey. Hamming “became very interested in the difference between those who do and those who might have done,” and he offered a number of answers to the question “why . . . so few scientists make significant contributions and so many are forgotten in the long run?” We have condensed Hamming's talk into the ten rules listed below:

## Rule 1: Drop Modesty

To quote Hamming: “Say to yourself: ‘Yes, I would like to do first-class work.' Our society frowns on people who set out to do really good work. But you should say to yourself: ‘Yes, I would like to do something significant.'”

## Rule 2: Prepare Your Mind

Many think that great science is the result of good luck, but luck is nothing but the marriage of opportunity and preparation. Hamming cites Pasteur's adage that “luck favours the prepared mind.”

## Rule 3: Age Is Important

Einstein did things very early, and all the “quantum mechanic fellows,” as well as most mathematicians and astrophysicists, were, as Hamming notes, “disgustingly young” when they did their best work. On the other hand, in the fields of music, politics, and literature, the protagonists often produce what we consider their best work late in life.

## Rule 4: Brains Are Not Enough, You Also Need Courage

Great scientists have more than just brainpower. To again cite Hamming: “Once you get your courage up and believe that you can do important things, then you can. If you think you can't, almost surely you are not going to. Great scientists will go forward under incredible circumstances; they think and continue to think.”

## Rule 5: Make the Best of Your Working Conditions

To paraphrase Hamming, what most people think are the best working conditions clearly are not, because people are often most productive when working conditions are bad. One of the better times of the Cambridge Physical Laboratories was when they worked practically in shacks—they did some of the best physics ever. By turning the problem around a bit, great scientists often transform an apparent defect into an asset. “It is a poor workman who blames his tools—the good man gets on with the job, given what he's got, and gets the best answer he can.”

## Rule 6: Work Hard and Effectively

Most great scientists have tremendous drive, and most of us would be surprised how much we would know if we worked as hard as some great scientists did for many years. As Hamming says: “Knowledge and productivity are like compound interest. Given two people with exactly the same ability, the one person who manages day in and day out to get in one more hour of thinking will be tremendously more productive over a lifetime.” But, Hamming notes, hard work alone is not enough—it must be applied sensibly.

## Rule 7: Believe and Doubt Your Hypothesis at the Same Time

Great scientists tolerate ambiguity. They believe the theory enough to go ahead; they doubt it enough to notice the errors and faults so they can step forward and create the new replacement theory. As Hamming says: “When you find apparent flaws, you've got to be sensitive and keep track of those things, and keep an eye out for how they can be explained or how the theory can be changed to fit them. Those are often the great scientific contributions.”

## Rule 8: Work on the Important Problems in Your Field

It is surprising but true that the average scientist spends almost all his time working on problems that he believes not to be important and not to be likely to lead to important results. By contrast, those seeking to do great work must ask: “What are the important problems of my field? What important problems am I working on?” Hamming again: “It's that simple. If you want to do great work, you clearly must work on important problems. . . . I finally adopted what I called ‘Great Thoughts Time.' When I went to lunch Friday noon, I would only discuss great thoughts after that. By great thoughts I mean ones like: ‘What will be the impact of computers on science and how can I change it?'”

## Rule 9: Be Committed to Your Problem

Scientists who are not fully committed to their problem seldom produce first-class work. To a large extent, creativity comes out of the subconscious. If you are deeply immersed in and committed to a topic, day after day, your subconscious has nothing to do but work on your problem. Hamming says it best: “So the way to manage yourself is that when you have a real important problem you don't let anything else get the center of your attention—you keep your thoughts on the problem. Keep your subconscious starved so it has to work on *your* problem, so you can sleep peacefully and get the answer in the morning, free.”

## Rule 10: Leave Your Door Open

Keeping the door to your office closed makes you more productive in the short term. But ten years later, somehow you may not quite know what problems are worth working on, and all the hard work you do will be “sort of tangential” in importance. He (or she) who leaves the door open gets all kinds of interruptions, but he (or she) also occasionally gets clues as to what the world is and what might be important. Again, Hamming deserves to be quoted verbatim: “There is a pretty good correlation between those who work with the doors open and those who ultimately do important things, although people who work with doors closed often work harder. Somehow they seem to work on slightly the wrong thing—not much, but enough that they miss fame.”

In our view, Rule 10 may be the key to getting the best research done because it will help you to obey Rules 1–9, and, most importantly, it will foster group creativity [9]. A discussion over lunch with your colleagues is often worth much more than a trip to the library. However, when choosing your lunchmates (and, by implication, your institution), be on your toes. As Hamming says: “When you talk to other people, you want to get rid of those sound absorbers who are nice people but merely say ‘Oh yes,' and to find those who will stimulate you right back.”
